# Heart rate variability in physically active individuals: reliability and gender characteristics

**DOI:** 10.5830/CVJA-2011-018

**Published:** 2012-03

**Authors:** Takshita Sookan, Andrew J Mckune

**Affiliations:** Department of Biokinetics, Exercise and Leisure Sciences, School of Health Sciences, University of KwaZulu-Natal, Durban, South Africa; Department of Biokinetics, Exercise and Leisure Sciences, School of Health Sciences, University of KwaZulu-Natal, Durban, South Africa

**Keywords:** heart rate variability, sympathovagal balance, reliability

## Abstract

**Purpose:**

To evaluate the reliability of short-term recordings (five minutes) of heart rate variability (HRV) and the association between HRV and gender.

**Methods:**

HRV time- and frequency-domain parameters were calculated in 44 physically active students (21 males and 23 females) over four consecutive days. A Suunto t6 heart rate monitor was used to obtain inter-beat intervals (IBIs) that were then transferred to Kubios HRV analysis software. The relative reliability [intra-class correlation (ICC)] and absolute reliability, [typical error of measurement (TEM) and typical error of measurement as a percentage (TEM%)] of the HRV parameters were then calculated for day 2 versus day 3 and day 3 versus day 4, with day 1 being a familiarisation day. The following HRV parameters were calculated: (1) time domain: resting heart rate (RHR), R–R intervals (IBI), standard deviation of normal-to-normal intervals (SDNN), root mean square differences of the standard deviation (RMSSD), percentage of beats that changed more than 50 ms from the previous beat (pNN50); and (2) frequency domain: low-frequency normalised units (LFnu), high-frequency normalised units (HFnu), low-frequency to high-frequency ratio in normalised units (LF/HFnu). An analysis of variance (ANOVA) with Tukey *post-hoc* testing was performed to compare HRV parameters in males and females. Significance was set at *p* ≤ 0.05.

**Results:**

The ICCs for both relationship 1 and 2 indicated primarily good to excellent (> 0.8) relative reliability. The lowest value was found in the LF/HFnu ratio (ICC = 0.36) for males. Absolute reliability was low with TEM% greater than 10% for all HRV parameters, except IBIs. Females demonstrated better relative (higher ICCs) and absolute reliability (lower TEM and TEM%) compared to males for the frequency domain. The relative and absolute reliability for the time domains were similar except for SDNN where the absolute reliability was higher in males. ANOVA illustrated significant gender differences for the LF/HFnu ratio (41% higher in males, *p* = 0.003), HFnu (12% higher in females, *p* = 0.02) and IBI (21% higher in females, *p* < 0.0001).

**Conclusions:**

Short-term recordings of HRV over three consecutive days demonstrated a high relative reliability. However, a low absolute reliability indicated large day-to-day random variation in HRV, which would make the detection of intervention effects using HRV difficult in individual participants. Females were shown to have a higher parasympathetic modulation of HRV, which may indicate an underlying cardioprotective mechanism in females compared to males.

## Abstract

Heart rate variability (HRV) is recognised as a versatile and promising non-invasive marker of autonomic nervous system (ANS) modulation.^1^ Research into the use of HRV has increased in both clinical and research environments and over a broad spectrum of disciplines.^2^,^3^ However, in the disciplines of sport and exercise science, there is limited information available on the reliability of HRV measures, in particular related to new, commercially available equipment that coaches and athletes have access to. This fact, together with the extensive number of variables that alter HRV measures, make it difficult to compare HRV studies and develop a universal standard.^2^ While most studies agree that age is inversely associated with HRV,^4^ research is less consistent on the impact of gender, with studies demonstrating that HRV measures are either the same or differ considerably between the genders and may also be HRV parameter dependant.^5^-^8^

Heart rate variability reflects the changes in the interval between heart beat (R waves) over time. The time between one R wave and the next, in milliseconds, is termed the R–R interval or the interbeat interval (IBI).^9^ The ANS governs the IBIs via the sympathetic and parasympathetic pathways.^10^ The relative dominance of either pathway over the other represents an alteration in the sympathovagal balance which is reflected in IBI changes.^11^ Under normal resting conditions in healthy individuals, it has been suggested that the parasympathetic pathway is dominant, resulting in a high HRV,^9^ while lower HRV and poor health has been linked to increased sympathetic activity at rest.^5^,^12^ However, research has demonstrated that the age, physical activity status, gender and the HRV parameter examined are important factors to consider when examining HRV. 5-8

Research has identified the potential use of HRV for identifying healthy and diseased states. In particular, a significant relationship between the ANS, low HRV and cardiovascular mortality, including sudden cardiac death, has been reported.^4^,^13^ In addition, studies have shown that trained athletes have higher HRV compared to sedentary individuals, suggesting that exercise training can increase HRV in normal populations.^14^ The over-training syndrome is assumed to be the consequence of an imbalance between long-term, inappropriate, high training volume and too little time for regeneration.^15^,^16^ Alterations in the ANS have been presented as a mechanism underlying the signs and symptoms of the over-training syndrome.^17^ A study that examined ANS activity in several middle-distance runners suggested that heavy training shifted the cardiac autonomic balance toward a predominance of the sympathetic over the parasympathetic drive, which was represented by a decrease in HRV.^17^

Heart rate variability has the potential to be a useful monitoring tool in the fields of health, fitness and sports performance. However, currently, there are issues regarding the standardisation and reproducibility of HRV measurement, as there are many confounding factors that can influence HRV. These include factors such as mood, alertness, mental activity, gender and age.^3^,^6^,^18^ While the research relating to gender differences is controversial,^5^,^7^,^18^ the relationship between age and HRV has been well documented.^5^,^6^,^8^,^19^ Reduced HRV is associated with progressing age and with an increased risk of cardiac events in clinically disease-free patients.^14^

The development of wireless heart rate-monitoring equipment, which has the ability to record IBIs has provided athletes, coaches, scientists and medical practitioners with mobile and easy-to-use systems that allow for the analysis of HRV. The commercially available Suunto t6 heart rate system (Suunto; Vantaa, Finland) is one such instrument that is widely used for monitoring heart rate during exercise. The validity of the Suunto t6 in measuring IBI for determining HRV has been reported recently.^1^

While previous studies have examined the reliability of other commercially available devices (Polar S810) for measuring HRV,^1^,^2^ there is limited information on the reliability of the Suunto t6 for HRV measurement.^1^,^20^ Analysis of the IBIs to determine HRV can be performed using custom software such as Kubios heart rate variability software version 2.0 (Biosignal Analysis and Medical Imaging Group, Department of Physics, University of Kuopio, Kuopio, Finland).^20^,^21^ To date, there are few publications reporting the use of both the Suunto t6 and Kubios software together.^20^

The primary aim of this study was therefore to investigate the reliability of Kubios software HRV measures calculated from the Suunto t-6 IBIs in physically active individuals. Furthermore, we investigated whether there were any gender differences in HRV parameters.

## Methods

This study was conducted on 50 physically active young adults (males: *n* = 25; females: *n* = 25), although data analysis was performed on 21 males and 23 females due to exclusion criteria, discussed in the statistical analysis of the data (Table 1). There were significant differences between the genders in terms of height, mass and percentage body fat, but no differences in age, body mass index, waist-to-hip ratio or weekly physical activity levels (Table 1). Participation was voluntary, and written informed consent was obtained from all participants. The study was approved by the Institution’s Biomedical Research Ethics Committee (REF: BE111/010).

**Table 1:** Descriptive Data, Physical Characteristics And Physical Activity Level Of Males And Females (Mean ± Sd)

	*Males (n = 21)*	*Females (n = 23)*	p
Age (years)	21.17 (1.55)	19.75 (1.76)	0.45
Height (cm)	177.3 (9.09)	160.5 (6.49)	< 0.0001*
Mass (kg)	76.19 (14.69)	60.41 (9.64)	0.0001*
BMI (kg/m^2^)	24.03 (2.64)	23.41 (2.89)	0.467
Waist/hip ratio	0.88 (0.07)	0.85 (0.068)	0.123
Percent body fat	13.73 (4.53)	18.63 (3.77)	0.0004*
IPAQ (Kcals/week)	5828 (3806)	5491 (3751)	0.789

IPAQ = International Physical Activity questionnaire.

Participants were excluded from the study if they had experienced a cold or feverish illness in the month leading up to the study, were smokers, had a pre-existing heart condition either current or in the past, were pregnant, diabetic, had congestive heart failure, or acute or chronic renal disease, if they have a pacemaker, or were taking type 1A anti-arrhythmics (quinidine, procainamide, disopyramide or moricizine).

## Assessment of physical activity status

The International Physical Activity questionnaire (IPAQ) is a validated questionnaire primarily designed for population surveillance of physical activity among adults (age range 15–69 years).^22^ This questionnaire was used to classify the physical activity status of the participants in the week leading up to the first (day 1) testing day. The IPAQ requires the summation of duration (in minutes) and frequency (days) for different categories of physical activity.

Based on this information, MET-minutes/week are calculated with the MET minute scores being equivalent to kilocalories expended per week. The participants were then classified into low, moderate or high physical activity levels. On average, the participants in the study were classified as being in the moderate category, with an average of 5 828 kcals (Table 1) expended per week (833 kcal expended per day). Moderate is defined by the IPAQ guidelines as a pattern of activity done on three or more days, at least 20 minutes per day and described as vigorous-intensity activity, or five or more days of moderate-intensity activity and/or walking at least 30 minutes per day.

## Protocol

Testing was conducted within the human performance laboratory (HPL) at our institution. The temperature in the HPL was maintained at 22°C with 50% humidity. Participants were provided pre-test instructions the week before the testing to help control for factors that could alter heart rate variability readings. They were asked to avoid caffeine, eating, heavy physical activity, smoking and alcohol intake for the 10 hours preceding each laboratory visit. Each participant attended four testing sessions for four days in a row at the same time of the day. Testing was performed between 07:00 and 21:00.

On testing day 1, which counted as the familiarisation day, each participant read the information sheet provided on the study, signed a written informed consent, completed a pre-test questionnaire (IPAQ) and medical history questionnaire. The medical questionnaire examined cardiovascular, metabolic and respiratory disease history (personal and family) as well as risk factors and signs and symptoms for these diseases. In addition, participants provided information on their current medication and supplement intake.

Height, mass, waist and hip circumferences and three site skinfolds (females: tricep, supra-iliac and mid-thigh; males: chest, abdominal and mid-thigh) were then measured. Height and weight were recorded using a calibrated medical height gauge and balance scale (Detecto, Webb City, USA). A Harpenden skinfold calliper was used for skinfold measurements to calculate percentage body fat using the Jackson and Pollock equations.^23^,^24^ Body mass index (BMI) [mass (kg)/height (m^2^)] and waist-to-hip ratio (waist:hip circumferences) were calculated and the HRV measurement protocol was then followed.

## Heart rate variability measurement

The participants were fitted with the Suunto t6 heart rate monitor (HRM) (Suunto; Vantaa, Finland). The electrodes on the transmitter were wet with water and were placed on the chest against bare skin to ensure good skin contact. Participants were tested while lying supine with the total testing time lasting 20 minutes. This time was divided into 15 minutes of rest followed by a five-minute measurement of IBIs. The IBIs were then transferred to a laptop (HP ProBook) computer where the data were stored in the Suunto team manager software program (Firstbeat Technologies, Ltd; Jyvaskyla , Finland).

The data were then exported as a text file to the HRV analysis software (Kubios heart rate variability software version 2.0; Biosignal Analysis and Medical Imaging Group, Department of Physics, University of Kuopio, Kuopio, Finland) for analysis of the following HRV parameters (1) time domain: resting heart rate (RHR), R–R intervals (IBI), standard deviation of normal-to-normal intervals (SDNN), mean square root differences of the standard deviation (RMSSD), percentage of beats that changed more than 50 ms from the previous beat (pNN50); and (2) frequency domain: low-frequency normalised units (LFnu), high-frequency normalised units (HFnu), low-frequency to high-frequency ratio in normalised units (LF/HFnu).

The Suunto t6 HRM and Kubios program^20^ comply with guidelines recommended by the Taskforce of the European Society of Cardiology and the North American Society of Pacing and Electrophysiology standards for measurement of HRV.^4^ Before processing, the IBIs were manually corrected for ectopic/missed beats. There is currently no universal method for identifying and editing ectopic beats. The amount and type of editing of IBI data has different effects on various HRV indices.^25^ In the present study, manual editing or interpolation^25^ of the IBI intervals was performed using the following guidelines: if a significantly higher IBI (representing an ectopic beat) was noted, then that reading was deleted and the average of the two adjacent IBIs replaced the deleted one. If a significantly lower value (representing a missed beat) was noted, that IBI was deleted and replaced with the previous IBI. If the ectopic or missed beats exceeded 20% of a participant’s overall five-minute recording, the participant was not included in the analysis.^2^ This occurred in six participants (four males and two females), with the result that only 44 participants were included in the final analysis (*n* = 21 males and *n* = 23 females).

Once the IBIs were imported into the Kubios program, the software automatically analysed the HRV in both time and frequency domains. Power spectral analysis was performed using the autoregressive (AR) algorithm in accordance with the recommendations.^4^ The AR algorithm was used as it yields improved resolution, especially for short-term HRV measurements.^20^,^21^ This algorithm creates a power spectral analysis with distinct frequency bands, namely high frequency (HF), low frequency (LF) and very low frequency (VLF).

The LF component has been proposed as reflecting both sympathetic and parasympathetic effects on the heart and occurs in a band between 0.04 and 0.15 Hz. However, researchers noted that the low-frequency band is influenced by baroreceptor-mediated regulation of blood pressure and reflects predominantly sympathetic activity.^26^ The HF (0.15–0.4 Hz) band corresponds with respiratory sinus arrhythmia (RSA) and is said to reflect parasympathetic activity.^26^ Chemoreceptor processes, thermoregulation, and the renin–angiotensin system have been linked with the VLF band. We did not use data collected from the VLF range for this study.

## Statistical analysis

The data were summarised using routine descriptive statistics (mean ± SD). Reliability measures were determined for day 2 versus day 3 and day 3 versus day 4. Absolute and relative reliability of several HRV parameters was calculated using the procedures described by Hopkins.^27^ Hopkins has argued that the statistical analysis used in reliability studies should include observed values and confidence limits of the typical error. These measures are sufficient to characterise the reliability of a measure and they substantially enhance comparison of the reliability of tests, assays or equipment.^27^

Absolute reliability is the degree to which repeated measurements vary for individuals. This type of reliability is expressed either in the actual units of measurement or as a proportion of the measured values (dimensionless ratio).^28^ Absolute reliability was calculated using the typical error of measurement (TEM) and TEM as a percentage (TEM%), expressed as percentage of the mean score.^27^

Sport and exercise science reliability studies have rarely reported the separate analysis of homoscedastic and heteroscedastic data.^28^ These parameters show how the measurement error relates to the magnitude of the measured variable. When the amount of random error increases as the measured values increase, the data are said to be heteroscedastic. When there is no relation between the error and the size of the measured value, the data are described as homoscedastic.^27^ Homoscedastic errors are expressed in the actual units of measurement (TEM) but heteroscedastic data are measured on a ratio scale. With homoscedastic errors, the raw data are analysed with conventional parametric analyses, but heteroscedastic data are transformed logarithmically before analysis or investigated with an analysis based on ranks.^28^

In the present study, the HRV data for each parameter were examined using the technique described by Hopkins^27^ and were found to be homoscedastic. TEM and TEM% were calculated with 90% CI for day 2 versus day 3 and for day 3 versus day 4, using a spreadsheet downloaded from http://www.newstats.org. The TEM is also known in statistical terms as the coefficient of variation (CV).

Furthermore, for reliability studies, it has been suggested that relative reliability is presented together with absolute reliability.^28^ Relative reliability is the degree to which individuals maintain their position in a sample with repeated measurements and is represented in the form of correlation coefficients. In this study we used interclass correlations (ICC) with 95% CI.^27^ The main advantage of ICC over Pearson’s correlation is that it can be used when more than one retest is being compared with a test.^28^ Various categories of reliability are based on the ICC. An ICC above 0.8 is usually regarded as good to excellent reliability, whereas an ICC between 0.6 and 0.8 may be taken to represent substantial reliability.^3^

In order to determine whether there were any gender differences in the HRV parameters, the data for each HRV parameter for days 2, 3 and 4 were combined and analysis of variance (ANOVA), with a Tukey *post-hoc* testing was performed to compare males with females. The data were analysed with STATISTICA version 8.0 (Statsoft Inc, Tulsa, Oklahoma, USA) for any statistical significance (*p* ≤ 0.05).

## Results

A total of 44 participants with complete data sets were analysed. The outcomes of the reliability analysis are presented in Table 2 for day 2 versus day 3 and day 3 versus day 4.

## Typical error of measurement

The absolute reliability indices for the HRV parameters are shown in Table 2. The data presented here offer precision estimates for single measurements of Suunto t6 HRV and data for making decisions when monitoring changes or responses to interventions in individuals.

**Table 2:** Reliability Indices For Frequency And Time Domain HRV Parameters

*Day 2 vs day 3*				*Day 3 vs day 4*			
*HRV parameters*		*TEM*	*TEM (%)*	*ICC*	*TEM*	*TEM (%)*	*ICC*
LF/HFnu	Female	0.25 (0.20–0.36)	31.4 (25.1–45.2)	0.80 (0.59–0.91)	0.24 (0.19–0.35)	29.7 (23.5–43.4)	0.83 (0.63–0.92)
	Male	0.54 (0.41–0.78)	48.1 (36.5–69.5)	0.36 (0.08–0.69)	0.43 (0.33–0.62)	40.4 (31.0–58.3)	0.72 (0.41–0.88)
HFnu	Females	6.63 (5.13–9.38)	11.5 (8.9–16.2)	0.88 (0.74–0.94)	7.92 (6.13–11.21)	13.4 (10.3–18.9)	0.81 (0.61–0.92)
	Males	12.58 (9.62–18.17)	24.6 (18.8–35.5)	0.55 (0.17–0.79)	8.95 (6.85–12.93)	16.7 (12.8–24.2)	0.78 (0.53–0.90)
LFnu	Female	7.78 (6.26–10.38)	17.8 (14.3–23.8)	0.84 (0.70–0.92)	7.26 (5.84–9.69)	17.2 (13.9–23.0)	0.86 (0.74–0.93)
	Male	13.04 (10.41–17.70)	26.6 (21.2–36.1)	0.52 (0.19–0.74)	9.40 (7.50–12.76)	20.2 (16.11–27.4)	0.75 (0.54–0.88)
IBIs (ms)	Females	44.90 (35.21–61.98)	4.8 (3.8–6.6)	0.83 (0.67–0.91)	45.89 (35.99–63.34)	4.9 (3.9–6.9)	0.79 (0.60–0.89)
	Males	53.46 (40.90–77.20)	4.8 (3.6–6.9)	0.90 (0.79–0.95)	46.46 (35.54–67.09)	4.1 (3.2–5.9)	0.93 (0.85–0.96)
SDNN	Females	28.83 (22.30–40.80)	31.2 (24.1–44.1)	0.77 (0.54–0.90)	35.58 (27.52–50.36)	32.2 (28.8–52.7)	0.72 (0.45–0.87)
	Males	20.70 (15.83–29.89)	19.9 (15.2–28.8)	0.70 (0.40–0.87)	20.62 (15.78–29.78)	20.2 (15.4–29.1)	0.73 (0.45–0.88)
RMSSD	Females	17.29 (13.37–24.47)	20.5 (15.8–29)	0.92 (0.82–0.97)	20.12 (15.56–28.48)	22.6 (17.5–32)	0.91 (0.80–0.96)
	Males	19.12 (14.63–27.62)	18.7 (14.3–27.1)	0.83 (0.64–0.91)	20.53 (15.71–29.65)	20.3 (15.5–29.3)	0.79 (0.55–0.89)
pNN50	Females	9.56 (7.39–13.53)	19.1 (14.8–27)	0.84 (0.67–0.93)	8.80 (6.80–12.45)	18.7 (14.4–26.4)	0.87 (0.72–0.94)
	Males	7.55 (5.78–10.90)	14.2 (10.9–20.5)	0.86 (0.69–0.94)	7.07 (5.41–10.21)	13.2 (10.1–19.1)	0.87 (0.72–0.95)

HRV = heart rate variability. Frequency domain: LFnu = low-frequency normalised units, HFnu = high-frequency normalised units, LF/HFnu = low-frequency to high-frequency ratio in normalised units. Time domain: IBI = R–R intervals, SDNN = standard deviation of normal-to-normal intervals, RMSSD = root mean squared differences of the standard deviation, pNN50 = percentage of beats that changed more than 50 ms from the previous beat. Reliability: ICC = intra-class correlation and is expressed as a mean (95% CI), TEM = typical error of measurement, TEM% = typical error of measurement as a percentage, and both are expressed as means (90% CI).

Overall for both males and females, the results show that for most HRV frequency-domain parameters, the second comparison day, day 3 versus day 4 had lower TEM values compared to the first day, day 2 versus day 3. This slight decrease in the TEM could be due to a familiarisation effect.^3^ Females demonstrated better absolute reliability in the HRV frequency domain as demonstrated by lower TEMs. Specifically, the TEMs for day 3 versus day 4 were lower in females for the LF/HFnu ratio (116% lower), 90% lower for HFnu (day 2 vs day 3) and 68% lower for LFnu (day 2 vs day 3). The lowest typical error of measurement as a percentage (TEM%) was 11.5% and was found in the females for Hfnu (day 2 vs day 3).

Time-domain results had a low TEM% for IBIs (day 2 vs day 3: 4.8 males and females; day 3 vs day 4: 4.9 females and 4.1 males). Overall, the TEM% was relatively high in most HRV parameters, specifically for LF/HFnu (day 2 vs day 3: 31.4% females and 48.1% males; day 3 vs day 4: 29.7% females and 40.4% males). The TEM was similar for males and females for the time-domain parameters, specifically, RMSSD, pNN50 and the IBIs. However, the TEM for SDNN was 42% lower in the males (day 3 vs day 4).

## Interclass correlations

The interclass correlations (ICCs) for relationship 1 ranged between 0.36 and 0.88 for the AR frequency domains and 0.70–0.92 for the time domains (Table 2). For day 3 versus day 4, the ICCs ranged between 0.72 and 0.86 for the AR frequency domains and 0.72–0.93 for the time domains. The ICCs for both day 2 versus day 3 and day 3 versus day 4 indicated good to excellent (> 0.8) reliability correlations for IBIs and pNN50 from the time-domain results. RMSSD reliability was good to excellent with the exception of the males for day 3 versus day 4, and showed substantial reliability of 0.79. SDNN variable results depicted substantial reliability for both day 2 versus day 3 and day 3 versus day 4.

Reliability ICCs for the frequency domain were lower compared to the time domain. The ICCs in the frequency domain were between 0.36 and 0.88, with the lowest value for these correlations being the LF/HFnu ratio (ICC = 0.36) for males. It was found that overall the male participants had lower ICCs when compared to females for the same HRV parameter in the frequency-domain analysis.

The ICCs for most time-domain parameters were similar between the males and females, with the exception of the IBIs. Males displayed high IBI ICC values (day 2 vs day 3 = 0.90, day 3 vs day 4 = 0.93), which indicated higher relative reliability compared to the females (day 2 vs day 3 = 0.83, day 3 vs day 4 = 0.79). Time-domain ICCs that were similar between males and females were SDNN (day 3 vs day 4), males (0.73) and females (0.72) and pNN50 (day 2 vs day 3), males (0.86) and females (0.84).

## Gender differences

There were significant differences between males and females for resting HR (*p* < 0.0001), R–R intervals (*p* < 0.0001) and for the frequency-domain HRV parameters, HFnu (*p* = 0.020) and LF/HFnu ratio (*p* = 0.003) (Table 3). The female resting heart rate was 16% higher than that of the males (*p* < 0.0001), while the IBIs were 21% higher in the males (*p* < 0.0001). The LFnu (*p* = 0.087) and LF/HFnu ratio (*p* = 0.003) were 13 and 41% higher in the males, respectively, while the HFnu (*p* = 0.02) was 12% higher in the females. There were no significant differences between males and females in the HRV time-domain parameters.

**Table 3:** Comparison Of Mean (± Sd) Of Days 2, 3 And 4 Of Heart Rate Variability Data By Gender

	*Men*	*Women*	p
Resting HR (b/min)	55.09 (8.1)	65.84 (7.2)	< 0.0001*
IBIs (ms)	1126 (169)	927.7 (101)	< 0.0001*
LFnu	47.95 (17.93)	42.48 (18.43)	0.087
HFnu	52.02 (17.73)	59.17 (17.16)	0.020*
LF/HFnu	1.114 (0.710)	0.792 (0.522)	0.003*
SDNN	103.1 (36.13)	93.39 (57.55)	0.252
RMSSD	101.3 (43.75)	87.57 (59.44)	0.137
pNN50	53.35 (18.71)	48.46 (22.77)	0.182

## Discussion

Despite extensive use and research in both clinical and physiological settings, HRV analysis is still poorly supported by rigorous reliability studies.^3^ The purpose of the present study was to examine the reliability and gender characteristics of standard parameters of HRV from short-term (five-minute) laboratory recordings in physically active individuals. The main findings of this study were that the reliability of HRV recording over consecutive days varied depending on the specific reliability index used, the HRV parameter examined as well as the gender of the population. A comparison of HRV frequency-domain parameters for males and females demonstrated significant gender differences in the sympathovagal balance.

Although the definition of a categorical rating of relative reliability based on ICCs is still controversial,^3^ the results from the present study demonstrate substantial to excellent relative reliability for the majority of time- and frequency-domain HRV parameters when comparing measurements obtained from days 3 and 4. For most measurements, the ICC was above 0.80 (‘good’),^28^ indicating that the repeated measurements reflect mostly the true value of HRV parameters relative to random error. Females demonstrated a higher relative reliability than males for all frequency-domain parameters. The high ICC of these short-term recordings indicate a considerable consistency with time, similar to previous studies.^3^,^8^

However, absolute reliability, indicated by the TEM and TEM%, revealed the presence of a large random error in all HRV parameters, particularly for the males. Females demonstrated better absolute reliability (lower TEM) than males for all parameters, particularly those in the frequency domain. The TEM% also indicated a low absolute reliability due to the high values found specifically for the LF/HFnu ratio. These findings are similar to those of Pinna *et al.* who found a greater random error (TEM) in frequency domain measures.^3^

These results might place doubts on the use of HRV indices in assessing interventions or treatment effects in individual participants, specifically when examining male participants or clinical populations.^3^ Furthermore, these results place doubt on the use of HRV measurement for monitoring performance changes in well-trained athletes. The high TEM and TEM% indicated that HRV would be unable to detect small but significant changes (≤ 1%) in performance.^29^ Considering the absolute reliability was low (high TEM and TEM% values), the large random error found in HRV would require significant changes in HRV to occur to deem them meaningful.^29^

## Gender differences

Findings of research examining HRV gender differences in healthy individuals are conflicting.^5^-^8^ Research has demonstrated that HRV measures are either the same or differ considerably between the genders and may also be HRV parameter or age dependant,^5^-^8^ and therefore, further research has been advocated.^3^ Umetani *et al.*^5^ has shown that HRV (for all measures) is significantly lower in ‘young’ (10–29 years) females compared to their age-matched male counterparts. The gender differences subsequently decreased and then disappeared with age and at different rates for the HRV parameters. Their findings suggested a higher level of parasympathetic activity in males.^5^

Conversely, Ryan *et al.*^6^ concluded that vagal high-frequency power was higher in females. They suggested this difference was most apparent for young (20–39) and middle aged (40–64) females. Raemaeker *et al.* noted that the LFnu (sympathetic dominance) was higher in females, however, HFnu (parasympathetic dominance) was not significantly different between the genders.^7^ Sinnreich *et al.*^8^ observed that RMSSD and HF components (measures reflecting predominantly vagal activity) were small and non-significant, but that the LF/HF ratio (suggested to reflect sympathetic/parasympathetic balance) differed substantially due to greater VLF and LF power found in the male participants. They concluded that their results illustrated relatively higher sympathetic activity in men compared to women. Our findings demonstrate a similar gender distinction to that of Ryan *et al.*^6^ and Sinnreich *et al.*^8^ A higher level of parasympathetic activity was found in the female participants compared to the males. This was demonstrated by a lower LF/HFnu ratio and higher HF value in the females.

The finding of a higher HRV and contribution of the parasympathetic nervous system to HRV in females may help explain the overall protection of pre-menopausal women from coronary heart disease (CHD), coronary mortality and sudden cardiac death, compared to males in this age group.^8^,^13^ Research has demonstrated that a high HRV is associated with significant cardiovascular health. Although not measured in the present study, the effect of oestrogen on HRV and parasympathetic activity in females may be a key factor in this finding.

Research examining HRV in pre- and postmenopausal women found a significant difference in HRV.^26^ Postmenopausal women had a significantly reduced HRV, as demonstrated by a higher relative power of LF and LF/HF ratio, which was related to a decline in the level of oestrogen. The authors concluded that a decline in the level of oestrogen from pre-menopausal to postmenopausal status favours the shifting of autonomic balance towards sympathetic dominance.^26^ In support of this finding, research has demonstrated that physiological levels of oestrogen increase vagal tone and suppress sympathetic modulation of heart rate in females.^30^

A limitation of this study was that respiratory rate was not controlled when measuring the IBIs over the five-minute recording period. Research has shown that the reliability of HRV measures was increased when respiratory rate was regulated.^12^ Furthermore, part of intra-subject variability was also due to the natural change of HRV parameters that occurs under the influence of factors such as mood, alertness and mental activity, which is very difficult to control for in any study.^3^

## Conclusion

Heart rate variability measures are a popular, non-invasive tool used to monitor autonomic function. Short-term recordings are easy to perform and are suitable for both clinical and physiological research. However, the findings of the present study have demonstrated a high relative reliability but low absolute reliability for HRV. In particular, HRV random error was higher in males. For clinical or sport/exercise science practice, these results place in doubt the use of HRV indices for assessing small (< 5%) intervention or treatment effects, specifically when examining male or clinical populations.^3^

Furthermore, these results place doubt on the use of HRV measurement for monitoring performance changes in well-trained or elite athletes, who typically require assessment techniques that can detect physiological or performance changes below 1%.^29^ Finally, specific HRV parameters differed between males and females, indicating a greater parasympathetic modulation of HRV in females. This finding suggests a possible mechanism for why pre-menopausal females have a lower incidence of coronary heart disease compared to males in the same age group, as well as compared to postmenopausal women.
